# Sex differences in presentation of pheochromocytoma and paraganglioma

**DOI:** 10.3389/fendo.2025.1463945

**Published:** 2025-01-23

**Authors:** Nora Azin Ali, Jan Calissendorff, Henrik Falhammar

**Affiliations:** ^1^ Department of Molecular Medicine and Surgery, Karolinska Institutet, Stockholm, Sweden; ^2^ Department of Endocrinology, Karolinska University Hospital, Stockholm, Sweden

**Keywords:** pheochromocytoma, paraganglioma, adrenal medullary tumor, male, female, symptoms, signs, diagnosis

## Abstract

**Purpose:**

The aim of the study was to investigate sex differences in presentation of pheochromocytomas and paragangliomas (PPGLs).

**Methods:**

This is a retrospective cohort study including 183 patients with confirmed PPGL (females n=100, pheochromocytoma n=156) between year 2005 and 2023, attending Department of Endocrinology, Karolinska University Hospital, Stockholm. The collected data included the mode of presentation, symptoms, biochemical, genetic and histopathological test results.

**Results:**

The mean age at surgery/diagnosis was 54.9 ± 17.0 years. Sweating was more common in females compared to males (44% vs 23%, p=0.003), and also takotsubo syndrome (10% vs 0% p=0.002). Males, on the other hand, were more likely to experience pallor (16% vs 4%, p=0.009), and were more often diagnosed due to investigations of a suspected PPGL (31% vs 18%, p=0.039) although no difference was found in the classic triad (sweating, palpitations and headache). Left-sided pheochromocytoma was more common among males than females (48% vs 29%, p=0.009). No differences between sexes were found in biochemical, genetical or histopathological results, or presence of metastasis at diagnosis.

**Conclusions:**

The reported symptoms by patients with PPGL were generally similar between the sexes, except for pallor and sweating. Takotsubo syndrome was more common among females. More males with PPGL were found based on suspicion than females. Further research into sex differences in various aspects of PPGL should be pursued.

## Introduction

Pheochromocytomas (PCCs) are neuroendocrine tumors in the adrenal medulla formed by chromaffin cells that secrete catecholamines (1). Paragangliomas (PGLs), originating from chromaffin cells outside the adrenal medulla, are classified into sympathetic and parasympathetic types (2). The latter will not be further discussed.

Pheochromocytomas and paragangliomas (PPGLs) cause symptoms due to excess catecholamines, primarily affecting the sympathetic nervous system (2). The classic triad of headaches, palpitations, and sweating occurs in only 17% of cases (3). Other symptoms are anxiety, constipation, syncope, and hyperglycemia.

Most PPGL cases have unknown causes, but about 30-40% are linked to genetic syndromes, with specific gene variants affecting the biochemical phenotype (4, 5). Some cases are found during screening in subjects with known gene variants.

The biochemical phenotype and amount of catecholamine secretion affects the clinical manifestation and course of the disease (6). The noradrenergic phenotype which predominantly produces norepinephrine, is more frequently found in paragangliomas. This phenotype typically presents with fewer catecholamine-related symptoms and are usually non-episodic (7). Sustained hypertension is the most common clinical manifestation of this phenotype (7). It is also associated with a higher risk of metastasis (6). The adrenergic phenotype which primarily produces epinephrine, is more likely to cause paroxysmal symptoms such as hypertension, palpitations, anxiety, and sweating. This phenotype is also linked to higher risk of hyperglycemia and hyperlipidemia (7).

Genetic variants are also an important factor. Different genetic variations are associated with specific biochemical phenotypes, affecting the clinical presentation as described (5). Hereditary pheochromocytoma and paraganglioma are typically diagnosed at a younger age and carries a higher risk of malignancy, particularly in cases involving variants in the *SDHB* gene (5, 8). Research has shown that tumor size correlates with catecholamine concentrations and the risk of metastasis (6). Other indicators of metastatic disease may include elevated chromogranin concentrations and extra-adrenal localization (5, 6).

Sex differences in symptoms related to excess catecholamines in patients with PCCs were first reported in 2008 by Lai et al. (9). Their study concluded that there was a significant difference in symptoms with females experiencing more physical and emotional symptoms than males (9). However, there have been no further reports on the differences in PPGL symptoms between males and females.

Thus, the aim of this study was to investigate sex differences in presentation of patients with PPGLs.

## Materials and methods

This is a retrospective single center study of patients attending the Department of Endocrinology, Karolinska University Hospital in Stockholm, Sweden. Electronic medical records from all patients with PPGL were reviewed manually to collect data. The inclusion criteria were patients with a PPGL and admission or attendance to the clinic between June 2005 and June 2023. To find the cases, all medical records with a code according to the international classification of disease version 10 (ICD10) of adrenomedullary hyperfunction (E.27.5) and/or malignant neoplasm of medulla of the adrenal gland (C74.1) were reviewed. Patients with unconfirmed PPGL were excluded. Patients who had insufficient data were also excluded. Patients with relapse of their PPGL were included only for the first episode.

The following parameters were collected for the current study: sex, age at surgery or in the unlikely event of no surgery age at diagnosis, tumor size, tumor side (left/right), histopathology, genetic results, blood pressure, mode of presentation (incidentaloma, PPGL suspicion, screening), reason for imaging, hypertension (sustained or paroxysmal), symptoms, other comorbidities such as diabetes mellitus type 2, cardiovascular disease, metastases, and biochemical evaluation.

The following biochemical parameters were analyzed for sex differences: 24h urinary (U)-epinephrine, U-norepinephrine, U-dopamine, U-metanephrine, U-normetanephrine, fasting plasma (fP)-metanephrine, fP-normetanephrine, fP-metoxytyramine, fP-chromogranin A, fP-glucose, random P-glucose and HbA1c. To facilitate comparisons and since not all individuals had all biochemical parameters available and different methods had been used, the number of times above the upper limit of normal (ULN) was calculated for catecholamine concentrations and their metabolites. This was calculated for the highest ULN for epinephrine/metanephrine, norepinephrine/normetanephrine, highest catecholamine ULN and chromogranin A. Moreover, those patients with only norepinephrine/normetanephrine secretion were identified.

The method used for measuring 24-hour urinary epinephrine, norepinephrine and dopamine was high-performance liquid chromatography (HPLC) (normal <80, <400 and <2600 nmol/24 h, respectively). For measuring fP-metanephrine, fP-normetanephrine and fP-metoxytyramin liquid chromatography tandem mass spectrometry (LC/MS/MS) was applied (normal <0.3, <0.6 nmol/L and <0,2, respectively). fP-chromogranin A was measured using enzyme-linked immunosorbent assay (ELISA) (normal < 3 nmol/L). Further details on the Methods can be found elsewhere (3, 10).

### Statistical analysis

The cohort was divided into two groups, females and males. For continuous parameters mean ± S.D was calculated for normally distributed variables otherwise median and range. Categorical parameters were summarized and presented as numbers and percentages. Continuous parameters were compared using student t-test or Mann-Whitney U-test as appropriate, while Fisher’s exact test was used for categorical parameters. Logistic regression analyses were done on the main symptoms and signs that differed between the sexes adjusted for age, tumor size, hypertension, and diabetes type 2 and are presented as odds ratios (ORs) with 95% confidence interval (95%CI). The statistical significance was set at P<0.05. R-Studio was used for all the statistical analysis (Vienna, Austria. URL: https://www.R-project.org/).

## Results

This study included in total 183 patients, of whom 100 (55%) were females and 83 (45%) were males. The details of the patients’ characteristics can be seen in **Table 1**. The mean age at surgery/diagnosis was 54.9 ± 17.0 years. The vast majority were patients with PCC (85%). There was a tendency of higher prevalence of PCC in the male group than the female group. Among patients with PCC, there were more males than females that had left-sided tumors (48% vs 29%, p=0.009). There was no difference in metastatic PPGL at surgery/diagnosis between the sexes. KI67 index tended to be higher among females.

**Table 1:** Characteristics of patients with pheochromocytoma or paraganglioma, also comparing females and males.

	All (n=183)	Females (n=100)	Males (n=83)	P-value (F vs M)
Age at sx/dx (years)	54.9 ± 17.02	54.6 ± 18.6	55.2 ± 15.05	0.806
Pheochromocytoma (n)	156 (85%)	81 (81%)	75 (90%)	0.095
Paraganglioma (n)	27 (15%)	19 (19%)	8 (10%)	0.095
PCC Left-sided tumor (n)	69 (38%)	29 (29%)	40 (48%)	0.009
PCC Right-sided tumor (n)	80 (44%)	47 (47%)	33 (40%)	0.370
PCC Bilateral tumors (n)	5 (3%)	3 (3%)	2 (2%)	1
Tumor size (mm)	44 (29-60)	48 (28.5-61.5)	40 (29.3-60)	0.602
KI67 (%)	1.0 (1.0-2.2)	1.1(1.0-2.5)	1.0 (1.0-2.0)	0.055
Metastasis of PPGL at sx/dx	3 (2%)	2 (2%)	1 (1%)	1
Total symptoms (n)	3 (1-5)	3 (1-5)	2 (1-4)	0.226
Systolic blood pressure (mmHg)	150.2 ± 30	149.4 ± 30	151.1 ± 30.4	0.692
Diastolic blood pressure (mmHg)	87 ± 15.7	87.2 ± 16.7	86.7 ± 14.5	0.849
Type 2 diabetes (n)	45 (25%)	28 (28%)	17 (20%)	0.301
Pre-diabetes^a^ (n)	30 (16%)	14 (14%)	16 (19%)	0.423
Cardiovascular disease pre dx (n)	54 (30%)	32 (32%)	22 (27%)	0.515
Takotsubo syndrome (n)	10 (5%)	10 (10%)	0 (0%)	0.002*
AMI (n)	17 (9%)	9 (9%)	8 (10%)	1
Stroke/TIA (n)	17 (9%)	11 (11%)	6 (7%)	0.450
Arrythmia (n)	11 (6%)	4 (4%)	7 (8%)	0.229
Preexisting hypertension (n)	102 (56%)	59 (59%)	43 (52%)	0.739
Other cancers^b^ (n)	23 (13%)	10 (10%)	13 (16%)	0.270
Hypothyroidism (n)	7 (4%)	6 (6%)	1 (1%)	0.129

*p<0.05.

a Pre-diabetes defined as HbA1c 42-47 mmol/mol or fGL 6-6.9 mmol/L.

b ”Other cancers” include colon cancer, breast cancer, rectal cancer, renal cell carcinoma, transitional cell carcinoma, prostate cancer, medullary thyroid cancer, myeloma, bladder cancer, chronic lymphocytic leukemia, gastrointestinal stromal tumor.

Age at sx/dx, Age at surgery/diagnosis; PCC, pheochromocytoma; AMI, acute myocardial infarction; TIA, transient ischemic attack.

The patients with PPGL had several previous comorbidities, with hypertension with antihypertensive treatment (56%), cardiovascular disease (30%), and type 2 diabetes (25%) being the most prevalent. The most common cardiovascular disease in the cohort were acute myocardial infarction (AMI) (9%) and stroke/transient ischemic attack (9%), with similar findings in females and males. However, in the female group, 10% had confirmed takotsubo syndrome (TS), whereas none were found in the male group (p=0.002). There were no significant differences between males and females in the other comorbidities.

**Table 2** describes the different ways in which PPGL was detected in patients. The most common way of discovery was as an incidentaloma. This was also the most common mode of presentation in the female group (73%) and in the male group (61%). The second most common way of discovery was through suspected PPGL (24%). Suspected PPGL was more common among males than females (31% vs 18%, p=0.039). The most common symptoms in the suspected PPGL group were palpitations (77%), consistent hypertension (75%) and paroxysms hypertension (60%). In this group, paroxysmal hypertension was more common among males than females (73% vs 39%, p=0.032), there were however no differences in the classic triad or other symptoms. The most common reasons for undergoing imaging were pain (26%) including abdominal, thorax or spine pain, abdominal pain (23%) or other abdominal symptoms (27%). There were no significant differences between males and females regarding the reason for imaging. The least common way of discovery was via screening due to a known genetic syndrome (7%).

**Table 2:** Mode of presentation and reason for imaging in patients found to have pheochromocytoma or paraganglioma, also comparing females and males.

	All (n=183)	Females (n=100)	Males (n=83)	P-value (F vs M)
Incidentaloma (n)	124 (68%)	73 (73%)	51 (61%)	0.110
Suspected PPGL (n)	44 (24%)	18 (18%)	26 (31%)	0.039*
Screening^a^ (n)	12 (7%)	7 (7%)	5 (6%)	1
Reason imaging pain^b^ (n)	48 (26%)	30 (30%)	18 (22%)	0.239
Reason imaging abdominal pain (n)	42 (23%)	26 (26%)	16 (19%)	0.296
Reason imaging abdominal symptoms (n)	49 (27%)	28 (28%)	21 (25%)	0.739
Reason imaging lung lx (n)	6 (3%)	5 (5%)	1 (1%)	0.223
Reason imaging trauma	4 (2%)	2 (2%)	2 (2%)	1

*p<0.05.

a Screening due to known genetic syndrome.

b Abdominal, thorax or spine pain.

PPGL, pheochromocytoma and paraganglioma; lung lx, lung investigation.

**Table 3** summarizes the symptoms and signs at diagnosis and compares females and males. The most reported symptoms in the cohort were consistent hypertension (58%), paroxysms of any symptom (52%) and palpitations (46%). In the male group 34% reported hypertension paroxysms, as compared to 22% in female group (p=0.096). Additionally, male patients experienced more pallor than females (16% vs 4%, p=0.009). Sweating was the only symptom that was more frequently reported by females than males (44% vs 23%, p=0.003). Sweating was non-significantly increased in premenopausal females compared to males (33% vs. 23%, p=0.313) but was in postmenopausal females compared to males (43% vs. 23%, p=0.034). The classic triad (sweating, palpitations, and headache) was experienced by 20% of the females and 16% of the males, which was a non-significant difference.

**Table 3:** Reported symptoms and signs of patients at diagnosis of pheochromocytoma or paraganglioma, also comparing females and males.

	All (n=183)	Females (n=100)	Males (n=83)	P-value (F vs M)
Paroxysms of any symptom (n)	95 (52%)	57 (57%)	38(46%)	0.140
Hypertension consistent (n)	107 (58%)	61 (61%)	46 (55%)	0.456
Hypertension paroxysms (n)	50 (27%)	22 (22%)	28 (34%)	0.096
Headache (n)	56 (31%)	32 (32%)	24 (29%)	0.748
Palpitation (n)	84 (46%)	47 (47%)	37 (45%)	0.768
Sweating (n)	63 (34%)	44 (44%)	19 (23%)	0.003*
Pallor (n)	17 (9%)	4 (4%)	13 (16%)	0.009*
Psychological symptoms^a^ (n)	64 (35%)	36 (36%)	28 (34%)	0.758
Flush (n)	25 (14%)	13 (13%)	12 (15%)	0.831
Nausea (n)	29 (16%)	17 (17%)	12 (15%)	0.688
Weight loss (n)	24 (13%)	17 (17%)	7 (8%)	0.123
Tiredness (n)	38 (21%)	24 (24%)	14 (17%)	0.275
Tremor (n)	20 (11%)	8 (8%)	12 (15%)	0.234
Dizziness/orthostatism (n)	30 (16%)	17 (17%)	13 (16%)	0.844
Classic triad^b^ (n)	33 (18%)	20 (20%)	13 (16%)	0.563
Abdominal pain (n)	4 (2%)	1 (1%)	3 (4%)	0.331
Abdominal symptoms (n)	11 (6%)	7 (7%)	4 (5%)	0.757
Constipation (n)	6 (3%)	5 (5%)	1 (1%)	0.223

*p<0.05.

a Psychological symptoms includes anxiety and panic attacks.

b Classic triad includes sweating, palpitation and headache.

**Table 4** presents the biochemical results. There were no significant differences found between males and females in the concentrations of catecholamines and their metabolites. Females and males had the same level of median U-epinephrine increase (1.5 ULN). The increase of median U-norepinephrine was 2.0 ULN for females and 1.6 ULN for males. There was no increase above ULN for U-dopamine or P-methoxytyramine in neither the female nor the males group. As for metanephrine and normetanephrine there were clear increases above ULN for both plasma and urine samples. Highest median ULN was 13.3 for females and 6.8 for males. For P-glucose and HbA1c there were no differences between males and females.

**Table 4:** Biochemical results of catecholamines and their metabolites, chromogranin A, plasma glucose and HbA1c in patients diagnosed with pheochromocytomas or paragangliomas, also comparing females and males.

	All (n=183)	Females (n=100)	Males (n=83)	P-value (F vs M)
U-E (ULN)^a^	1.5 (0.6-3.6)	1.5 (0.4-3.5)	1.5 (0.8-5-2)	0.223
U-NE (ULN)	1.7 (0.9-5.1)	2.0 (0.9-6.3)	1.6 (1-3.8)	0.749
U-D (ULN)	0.7 (0.5-1)	0.7 (0.5-1)	0.8 (0.5-1.1)	0.986
fP-ME (ULN)	2.7 (0.9-12.8)	2.7 (0.8-12.5)	3 (1-12.9)	0.632
fP-MNE (ULN)	7 (2.8-21.7)	10.3 (3-26-7)	5.9 (2.9-17.6)	0.361
U-ME^b^ (ULN)	4.8 (1.2-17.3)	28.5 (17.5-39.6)	1.9 (0.6-7.8)	0.240
U-MNE^b^ (ULN)	2.1 (1.3-3.1)	6.8 (4.7-8.9)	1.4 (1.1-2)	0.267
fP-Metoxytyramin (ULN)	0.5 (0.5-1.1)	0.7 (0.5-1.6)	0.5 (0.5-0.6)	0.198
E/ME highest (ULN)	2.7 (1-13.3)	2.6 (0.8-12.5)	3 (1.3-14)	0.358
NE/MNE highest (ULN)	5.7 (2.7-18.3)	8.2 (2.2-22.5)	4.6 (2.8-12.9)	0.318
Only NE/MNE secretion	47 (26%)	29 (29%)	18 (22%)	0.309
Highest (ULN)^c^	10.3 (3.7-26.8)	13.3 (3.7-29.3)	6.8 (3.8-23.7)	0.323
fP-ChrA (ULN)	2.7 (1.1-5)	2.9 (1-6.2)	2.5 (1.1-3.9)	0.390
fPGL (mmol/L)	6.1 (5.4-6.8)	6.4 (5.5-7.1)	6.1 (5.4-5.9)	0.187
Random PGL (mmol/L)	7.4 (6-31)	6.5 (6-12.3)	8.3 (6-10.2)	0.835
HbA1c (mmol/mol)	43 (40-52)	43 (40.3-55.8)	43 (39-46)	0.343

a U-E (ULN) calculated by dividing U-E by the upper limit of normal (ULN).

b The levels of catecholamine metabolites in urine was measured only in six patients.

c The highest increase of catecholamines above the upper limit of normal.

U-E, U-epinephrine; U-NE, U-norepinephrine; U-D, U-dopamine; fP-ME, fasting plasma (fP)-methoxy epinephrine; fP-MNE, fP-methoxy norepinephrine; fP-ChrA, fP-chromogranin A; fPGL, fasting plasma glucose.

**Table 5** summarizes the genetic testing. There were no differences between males and females regarding genetic testing and results. Overall, 18% had tested positive for one or multiple genes associated with PPGL. Negative results for one or multiple genes associated with PPGL was seen in 44% of the patients. Patients who had no gene results available at the time of data collection were 38%, however, some of them were waiting on results of whole genome sequency performed in another study. If considering only those who underwent gene testing, 29% were positive. Positive results for MEN2A (*RET*), neurofibromatosis type 1 (*NF1*) and succinate dehydrogenase (*SDHx*) variants were equally common between the sexes.

**Table 5:** Genetic screening results in patients diagnosed with pheochromocytomas or paragangliomas, also comparing females and males.

	All (n=183)	Females (n=100)	Males (n=83)	P-value (F vs M)
Positive gene variant	33 (18%)	18 (18%)	15 (18%)	1
Negative gene variant/gene panel^a^	81 (44%)	46 (46%)	35 (42%)	0.655
Negative gene panel^b^	60 (33%)	33 (33%)	27 (33%)	1
Not investigated^b^	69 (38%)	36 (36%)	33 (40%)	0.647
*MEN2A* positive	10 (5%)	5 (5%)	5 (6%)	0.758
*NF1* positive	10 (5%)	4 (4%)	6 (6%)	0.516
*SDHx* positive	8 (4%)	5 (5%)	3 (4%)	0.730

a Includes test for multiple genes, the number of genes has changed over the years.

b Includes patients that no genetic results were available, however, some of them were waiting on the results of whole genome sequency performed in another study.

*MEN2A*, multiple endocrine neoplasia type 2; *NF1*, neurofibromatosis type 1; *SDHx*, succinate dehydrogenase.

The results of the logistic regression analysis are presented in **Table 6**. The analysis indicates that females had a positive association with the symptom of sweating (OR 2.65, 95%CI 1.4-5.14, p=0.003). Males showed a positive association with the symptom of pallor (OR 4.46, 95%CI 1.51-16.35, p=0.012). These findings remained significant after adjusting for the factors age, tumor size, hypertension, and type 2 diabetes. A logistic analysis for TS could not be performed since there were no males in the study with TS.

**Table 6:** Logistic regression analysis of main results of 183 patients with pheochromocytomas or paragangliomas.

	Unadjusted results			Adjusted results*		
	OR	95% CI	P-value	OR	95% CI	P-value
Sweating F vs M	2.65	1.4 - 5.14	0.003**	2.62	1.37 - 5.15	0.004**
Pallor M vs F	4.46	1.51-16.35	0.012**	5.27	1.71 – 20.2	0.007**

*Adjusted for age, tumor size, hypertension, and diabetes type 2. **p<0.05 F, female. M, male. Logistic regression analysis was not possible for takotsubo syndrome since no male had this complication but was significant when doing Fisher’s exact test.

## Discussion

This study is the first comprehensive study to investigate the differences in how PPGL presents in females and males. Generally, the reported symptoms were similar between sexes, but pallor being more common in males and sweating more common in females. These findings persisted even after adjusting for other factors. More males with PPGL were found based on suspicion and left-sided PCC was more common among males while TS was more frequent among females. There was no difference in classic triad between the sexes. Of the three symptoms that make up the classic triad, sweating was more prevalent among females while the other two symptoms, palpitations and headache, were similar between sexes.

Lai et al. published the first report that identified sex-based differences in symptoms of patients with PCC. In the study females reported more symptoms such as headaches, dizziness, anxiety, tremors, weight changes, numbness, and changes in energy levels, along with a higher overall symptom score. However, our study found no sex-based differences in total symptom score, headaches, dizziness, psychological symptoms or tremors. We did not evaluate numbness and changes in energy levels. Regarding weight changes, we only assessed weight loss, which was reported non-significantly more frequently by females. Our study only found sex differences in pallor and sweating. Although Lai et al. showed that females more commonly reported these symptoms, no significant difference between the sexes was found (9).

Several factors may explain the variations in results. The primary difference lies in the study design. Lai et al. used a self-reporting questionnaire before the diagnosis, including only 58 patients who were all patients with PCC (9). Also, their cohort was nearly 10 years younger than our patients which may be an effect of them having been referred to National Institutes of Health (NIH), i.e., it can be assumed that there were some biases. The total symptom score difference may be due to differences in the symptoms evaluated and their quantity. Moreover, the comorbidities were not described by Lai et al. and may have influenced the outcome. Our retrospective data collection from electronic medical records in Sweden contrasts with the NIH’s location in the US, potentially introducing cultural differences in symptom reporting.

There are symptoms and conditions that are generally more common among females than males. For instance, females are more likely to suffer from headaches, especially migraines, which occur twice as often in females. Females who suffer from migraines also report more severe symptoms than men (11). Females are also more prone to experience anxiety and panic attacks (12). These sex differences, which could also be caused by PPGL, were interestingly not observed in our study.

Sweating can result from various conditions and was found in this study more commonly among females. No differences were found in the prevalence of other common conditions that may cause sweating, such as cardiovascular disease, hyperthyroidism, other cancers, and diabetes (13, 14). However, it is important to consider menopause when interpreting this finding. Menopause typically occurs between ages 45 and 55 and lasts about seven years (15, 16). In the current study 33% of the females who had surgery or were diagnosed with PPGL were within the age range of menopause. Vasomotor symptoms, such as flushes and sweating are experienced by around 75% of females during menopause (17). This could be the explanation of the increased reported sweating in the females of this study. However, flushing was not more frequent among females. Therefore, PPGL may be the cause of this sex difference in sweating. On the other hand, women with PPGL might underreport symptoms, attributing them to menopause instead. In this study, sweating was particularly more common in postmenopausal women than in males. It was also more prevalent among premenopausal women, although this difference was not significant. These findings indicate that menopause is unlikely to have an extensive impact on the results. It remains unclear why sweating was more common among females with PPGL, and this study alone cannot explain it.

Pallor, characterized by paleness of the skin, was in this study more commonly experienced by males than females. Anemia is a common cause of pallor, but there was no difference in its prevalence between the sexes in this study. One could speculate that males may be more exposed to triggers for pallor such as physical activity or alcohol consumption, although data on these factors were not available. Another possible explanation is that menopausal flushes could mask females’ pallor.

Pallor being more common among males could also be due to differences in adrenergic receptor activity. There is a study that describes higher alpha-receptor sensitivity among males, resulting in more vasoconstriction compared to females. This higher alpha-receptor activity and/or density may explain why pallor is more common among men (18). Women generally exhibit greater sensitivity to the vasodilatory effects of beta-receptors, which can counteract the vasoconstrictive effects of alpha-receptors (19).

In our study, 56% of participants were receiving hypertension treatment before being diagnosed with PPGL. This aligns with the 58% of patients who had consistent hypertension. However, a study by Yu et al. reported that 70% of PPGL patients had a hypertension history (20). Their single-center study in China may not be directly comparable to ours. They also noted a higher prevalence of PGL, which can lead to more consistent hypertension, potentially aiding in its diagnosis as hypertension (20).

In the current study, males tended to have more paroxysmal hypertension. Catecholamine levels and biochemical phenotype may reflect whether the symptoms are constant or paroxysmal. PGL typically exhibit a noradrenergic phenotype, whereas PCC commonly have an adrenergic phenotype (4). PGL often produce lower and more consistent levels of catecholamines compared to PCC. This results in patients with PGL being more likely to experience sustained high blood pressure while patients with PCC have more episodic high blood pressure (4).

The higher prevalence of paroxysmal hypertension in males in this study may be due to the higher percentage of PCC in the male group, albeit this did not quite reach significant levels. The biochemical results for catecholamines also showed a small non-significant indication of this. There was a slight non-significant difference in the NE/MNE highest, which was found to be higher in females, indicating a possible tendency toward a more noradrenergic phenotype among females. Tumors only secreting NE/MNE were also non-significantly more common in females, supporting this trend. Other factors that may have influenced the outcomes include pre-existing hypertension, treatment with anti-hypertensive medication, and potential triggers for attack like physical activity, anxiety and certain medications (21).

The most common way that PPGL was detected in this cohort was as incidentalomas. This finding is more frequent than detecting them based on symptoms, which reflects the increased use of imaging in clinical practice today (3). This shift may also explain why the classic triad of symptoms is not as commonly observed in both sexes, as more are found as incidentalomas (3). We consider this trend beneficial as it helps identify PPGL cases that may otherwise go unnoticed and untreated, potentially leading to fatal outcomes over time. A study from Sweden conducted in the 1980s found that 40% of patients with PPGL were diagnosed post-mortem (22). Identifying and treating PPGL is critical due to its potential for malignancy and the high risk of cardiovascular complications and mortality (2).

This study found that males were more likely to be investigated for a suspected PPGL than females. Diagnosing PPGL is challenging due to its wide range of symptoms that can resemble other diseases (23). Patients with the classic symptoms are often suspected of having PPGL. In the suspected group paroxysmal hypertension was more common among males. However, our study shows that the classic triad was equally frequent in both sexes, even in the suspected group alone. Thus, the classic triad cannot explain the higher number of suspected male cases. Menopausal women may underreport symptoms, concealing potential PPGL signs. Unfortunately, we did not have data available on menopausal status nor estrogen replacement therapy in this study. PPGL symptoms may also be misdiagnosed as common conditions, such as migraine and anxiety, which are more prevalent in females than males (11, 12). This could also be a contributing factor to the fact that more men are diagnosed based on suspicion.

Females tend to seek healthcare services more often than males, but this does not guarantee that they receive the same level of attention as males do. In conditions like ACS and stroke, females have a higher risk of delayed diagnosis (24, 25). They are less likely to be diagnosed with definite TIA/stroke despite exhibiting similar symptoms as males (26). Males often receive earlier diagnosis for certain cancers, despite females reporting poorer health and seeking care more frequently (27). It is possible to speculate a similar trend among patients with PPGL, suggesting that men may be taken more seriously and more often suspected of having the disease.

TS can be caused by several stress factors, including PPGL catecholamines (28). This study found that TS was more common among females than males with PPGL, aligning with the fact that females account for 90% of TS cases overall (29). The general sex differences in TS are unclear, and this is also reflected in patients with PPGL. In this study, women generally had higher levels of catecholamines, but this difference was not significant. Additionally, there were no indications that women with PPGL were diagnosed later than men in this study.

Left-sided PCC tumors were significantly more common in males as compared to females. Bechmann et al. found that while adrenal tumors are generally more common in the left adrenal gland, PCC tumors are more common in the right adrenal gland (30). Moreover, the study revealed that left-sided neuroblastomas and adrenocortical carcinomas have higher rates of metastasis than right-sided tumors. They also suggested a link between left-sided PCC tumors and increased metastatic disease (30). However, our study found no difference between the sexes with regards to metastatic PPGL at diagnosis. The reason why adrenal tumors are more commonly found in left adrenal gland than the right may be due to detection biases arising from their anatomical differences. The left gland, surrounded by hypoattenuating retroperitoneal fat, positioned above the left kidney and not compressed by other structures, is easier to visualize. In contrast, the right adrenal gland, which has less surrounding fat and is located between the liver and kidney. This makes detecting tumors on the right side more challenging (31). The reason men in this study had more left-sided PCC than females is unclear.

Regarding the results of genetic testing, there were no differences between the sexes. In a small study of 56 patients with PCC, 50% of *NF1*-positive patients were males, similar to our findings (32). The overall prevalence of positive genes was only 18%. However, 38% of our cohort had no genetic results available at the time of the data retrieval process. Among those with available results, 29% had positive gene results, which aligns with previous studies (23).

## Strengths and limitations

This study has several strengths, including a fairly large number of patients with PPGL and focuses on an area with limited knowledge. Additionally, a large amount of different data was collected from each patient. A retrospective study has the inherited limitations of missing data and the information bias. While prospective studies are ideal, enrolling enough patients for such rare disease in a reasonable time is challenging. It would have taken 19 years to prospectively include the same number of patients. Most variables in this study are unlikely to differ significantly between retrospective and prospective designs. The study being a single center study restricts its generalizability. A multicenter prospective approach could have been beneficial, but a large number of centers would have been required to achieve the same number of patients in a reasonable time. We know from experience that asking actively for PPGL symptoms occurs during a patient’s first clinic visit to the endocrinologist (when the results of catecholamine concentrations are available). There is no standardized questionnaire used, which may lead to underreporting of symptoms. On the other hand, not having a questionnaire allows to focus on the symptoms that the patient is affected by the most. Males and females may express their symptoms different, but it should be the same with a prospective design. As previously mentioned, menopause and other common conditions may obscure the reporting of PPGL symptoms. Unfortunately, details on menopausal status and estrogen replacement therapy were unavailable. Additionally, the symptoms may be assessed differently by the interviewers, depending on the individual’s sex. It is challenging to predict how this might affect the results. One could speculate, based on findings from other conditions that have been extensively studied, that women’s symptoms may not be taken as seriously as men’s.

## Conclusion

This study is the first large assessment of sex differences in the presentation of PPGL. The reported symptoms in both females and males with PPGL were generally similar, except for pallor being more common in males and sweating more common in females. More males with PPGL were found based on suspicion than females, although no differences were found in the classic triad or biochemical markers. Left-sided PCC was more frequent among males and TS among females. Further research into sex difference in various aspects of PPGL should be pursued, e.g., with regards to disease progression, treatment and short- and long-term outcomes, and link them to the findings of the current study.

## Data Availability

The datasets presented in this article are not readily available because privacy and legal reason. Requests to access the datasets should be directed to Henrik Falhammar.
